# Risk factors for early eyelid swelling following blepharoptosis surgery: A retrospective study

**DOI:** 10.1016/j.jpra.2025.12.003

**Published:** 2025-12-11

**Authors:** Rieko Shimizu, Aiko Oka, Shiho Watanabe, Hiroko Ochiai

**Affiliations:** a Department of Plastic and Reconstructive Surgery, Keio University School of Medicine, Shinjuku-ku, Tokyo, Japan; b Department of Plastic and Reconstructive Surgery, NHO Tokyo Medical Center, Meguro-ku, Tokyo, Japan; c Department of Plastic and Reconstructive Surgery, International University of Health and Welfare Narita Hospital, Narita-shi, Chiba, Japan

**Keywords:** Blepharoptosis surgery, Eyelid swelling, Starling’s law, Postoperative management, Excess skin removal

## Abstract

**Background:**

Postoperative swelling of the eyelids surgery can cause discomfort and reduce patient satisfaction. This study is a retrospective analysis of factors associated with early postoperative swelling after blepharoptosis surgery.

**Methods:**

The study included 105 patients who underwent primary bilateral ptosis surgery performed by the same plastic surgeon between April 2020 and December 2022. Aponeurotic repair, excess skin removal and lateral horn release were performed as needed, depending on the clinical findings in each case. Postoperative swelling was rated on a four-point scale: 0 (no swelling), 1 (mild), 2 (moderate), and 3 (severe).

**Results:**

Postoperative swelling was significantly correlated in patients with a larger width of skin excision, diabetes, aging, hypertension, high intraoperative systolic blood pressure, and longer surgery duration. Detailed examination showed that the length of aponeurosis advancement and changes in margin reflex distance (MRD) did not significantly affect swelling.

**Conclusions:**

Starling’s law is important to consider in preventing postoperative swelling, as it explains which factors contribute to edema. Surgical manipulation also triggers factors that cause swelling. Bleeding, inflammation, and lymphatic damage further exacerbate swelling. Knowing the risk factors for swelling in advance helps predict postoperative outcomes and helps inform patients, which may also improve postoperative management. Future studies using multivariate analysis are expected to provide valuable insights into preventing and managing postoperative eyelid swelling.

## Introduction

Blepharoptosis is a condition primarily caused by myogenic, neurogenic, aponeurotic, mechanical, or traumatic factors.^1^ Various surgical techniques are proposed based on the underlying cause and aim to improve both visual function and aesthetics, thereby enhancing patients’ quality of life. However, as the eyelids are prone to swelling and are a highly visible part of the face, postoperative swelling can reduce patient satisfaction.

Postoperative eyelid swelling typically includes at least 24–48 h of pain and discomfort, but severe or prolonged swelling can lead to more serious issues. Specifically, severe swelling poses a risk of recurrent ptosis or recurrent eyelid laxity, which can diminish visual function and cause aesthetic problems.^2^^,^^3^ Additionally, prolonged swelling may promote tissue fibrosis and cause persistent disruption of local fluid homeostasis,^4^ leading to complications such as eyelid eversion or retraction, thereby putting the patient at risk of visual impairment.^5^ This poses a physical and mental burden on patients, increases healthcare costs and treatment times, and may require additional surgeries. To prevent these issues and implement appropriate preventive measures, the causes and risk factors of postoperative eyelid swelling should be identified.

Despite the urgency of postoperative swelling, few studies have examined the factors contributing to swelling after ptosis surgery. Therefore, this study aims to elucidate the mechanism of swelling by identifying risk factors for early postoperative swelling after bilateral blepharoptosis surgery. Specifically, we aimed to identify patient-related risk factors for postoperative swelling by evaluating preoperative and postoperative conditions, intraoperative parameters, and the postoperative management from multiple perspectives.

## Patients and methods

### Study design

This research was approved by the Institutional Review Board of the National Tokyo Medical Center (Study number R23-106, March 27, 2024) and conducted following the Declaration of Helsinki (2013). This retrospective study included 105 patients who underwent bilateral blepharoptosis surgery performed at the Department of Plastic and Reconstructive Surgery, National Hospital Organization Tokyo Medical Center between April 2020 and December 2022. The sample size was based on all eligible patients who met inclusion criteria during the study period. Patient conditions were defined as being primary surgery; patients undergoing surgery for the first time(#1–6), or acquired; patients in whom only excess skin excision was performed were also included. Cases with congenital ptosis, blepharospasm, or those requiring procedures other than aponeurotic repair, skin excision, or lateral horn release were excluded. The study design and reporting adhered to the STROBE (Strengthening the Reporting of Observational Studies in Epidemiology) guidelines. All the patients involved in this study provided an informed consent form after a detailed explanation of the benefits and risk of the procedure was provided.

We retrospectively reviewed the charts and required data pre- and postsurgery of the 105 patients. The following demographic data was collected: age at the time of surgery, gender, presence of underlying conditions (hypertension, diabetes, hyperlipidemia), use of antithrombotic medications, blood pressure during surgery, duration of surgery, width of skin excised, and length of aponeurosis advancement. In addition, photographs, the margin reflex distance (MRD), palpebral fissure height, and levator function, before the surgery and 1 week, 1 month, and 3 months postsurgery were collected.

## Methods

### Surgical technique

All surgeries were performed by a single plastic surgery surgeon, and the surgical procedures were standardized according to the following methods. Minor intraoperative modifications were made based on each patient’s anatomical features, such as the amount of pretarsal tissue removal or the depth of dissection, but the overall approach and sequence of surgical steps were consistent across cases. These variations, although within a standardized framework, may affect the degree of postoperative swelling. Systolic and diastolic blood pressures were continuously monitored and recorded during the surgery, and appropriate measures such as pausing the operation were taken if any abnormalities were observed.

The patients underwent surgery under local anesthesia using 1 % lidocaine in 1:100,000 epinephrine (Xylocaine® Injection 1 % with Epinephrine, Sandoz Pharma K.K, Tokyo, Japan).

The incision line was marked along the predetermined double-fold line, and the width of the skin excision was determined by measuring the amount of excess skin with a ruler. A portion of the skin was excised to expose the orbicularis oculi muscle, and a narrow strip of the muscle was removed to expose the anterior side of the tarsus, minimal excision of the pretarsal fibrofatty tissue was performed to expose the superior tarsal border. For patients requiring aponeurosis advancement, the aponeurosis was released from the caudal side, and the aponeurosis was then dissected free from the underlying Muller’s muscle. Next, the aponeurosis was separated along the loose areolar layer in a cranial direction, up to the level of the musculoaponeurotic junction. The advancement length of the levator aponeurosis was determined by an intraoperative adjustment technique: after the patient was placed in a supine or sitting position and asked to open and close their eyes, the sutures were adjusted appropriately. The advanced aponeurosis was fixed to the tarsus at three points by using absorbable thread (6-0 PDS® plus, Johnson & Johnson, New Jersey, USA). After confirming that complete eyelid closure was possible, the skin-aponeurosis sutures were placed as indicated in common double eyelid surgery. The duration of the surgery was also recorded.

### Postoperative care

The surgical region was immediately treated with an ointment containing fradiomycin sulfate and methylprednisolone. Soft compression was applied with a net bandage, and the patient was instructed to remain at rest overnight. The compression was released the following morning, and the patient was allowed to wash their face and then instructed to apply ointment to the wounds. The threads were removed approximately 1 week after surgery. Efforts were made to provide consistent postoperative care for all patients.

### Evaluation of swelling

**Figure 1:**
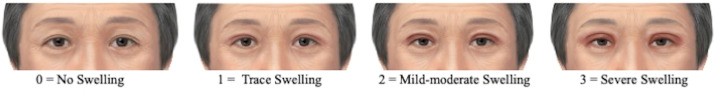
Clinical evaluation scale for postoperative eyelid swelling. The photos of patients were taken 1 week after surgery, and the severity of postoperative eyelid swelling was scored by two plastic surgeons on a scale of 0 (no swelling), 1(trace welling), 2(mild-moderate welling) and 3 (severe welling) according to the illustration.

Two plastic surgeons assessed the degree of swelling using preoperative and 1-week postoperative photographs obtained from the medical records. The evaluation method was determined based on the literature,^2^ and each grader rated the swelling of each eyelid on a four-point scale: 0: no swelling, 1: trace swelling, 2: mild-moderate swelling, or 3: severe swelling (Figure 1).

### Statistical analysis

To validate the relationship between eyelid swelling and patient characteristics, we analyzed the data on age, gender, the presence of underlying conditions (hypertension, diabetes, hyperlipidemia), and the use of antithrombotic medications using a nonpaired *t*-test. In addition, to evaluate the effect of the operation, regression analysis was conducted on the data for intraoperative blood pressure, duration of surgery, width of skin excised, length of aponeurosis advancement, and MRD change. All statistical analyses were performed using Statistical Package for the Social Sciences software version 30.0.0 (IBM, NY), and a *p*-value of < 0.05 was considered statistically significant.

## Results

### Patient background

A total of 105 patients and 210 eyelids were included in this study, comprising 30 males and 75 females. The average age was 73.57 years for males and 72.57 years for females, with no statistically significant difference between genders. The prevalence of comorbidities among the patients was as follows: hypertension 37.1 %, diabetes 18.1 %, and hyperlipidemia 11.3 %. Additionally, 9.5 % of the patients were on antithrombotic medications (Table 1).

**Table 1:** Basic characteristics of participants (*n* = 105).

Characteristics	Values
Age, years	72.86 ± 10.0 (42–91)
Sex (male: female),%	28.6: 71.4
Hypertension,%	37.1
Diabetes,%	18.1
Hyperlipidemia,%	11.3
Antithrombotic medications,%	9.5

### Surgery-related factors

On average, the width of the skin excision was 6.76 ± 3.36 (0–14) mm, and the length of aponeurosis advancement was 9.29 ± 3.59 (0–15) mm (Table 2). The surgical duration was 45.63 ± 11.88 (22–84) minutes, intraoperative maximum systolic blood pressure was 160.91 ± 21.73 (115–210) mmHg, intraoperative maximum diastolic blood pressure was 78.06 ± 14.23 (55–120) mmHg, and the MRD change from pre- to 3 months post-operation was 2.00 ± 1.22 (0–5) mm.

**Table 2:** Surgical metrics of *n* = 210 eyelids.

Surgical metrics	Mean values
Width of skin excision (mm)	6.76 ± 3.36 (0–14)
Length of aponeurosis advancement (mm)	9.29 ± 3.59 (0–15)
Surgical duration (min)	45.63 ± 11.88 (22–84)
Intraoperative max SBP (mmHg)	160.91 ± 21.73 (115–210)
Intraoperative max DBP (mmHg)	78.06 *±* 14.23 (55–120)
MRD change from pre- to 3 months post-operation (mm)	2.00 ± 1.22 (0–5)
Data expressed as mean ± standard deviation (range)	

### Association with postoperative eyelid swelling

As shown in Table 3, Table 4, 55.7 % of patients exhibited mild to moderate swelling, while 26.4 % showed severe swelling. The mean swelling score was 2.09 ± 0.66, with high inter-rater reliability, supporting the assessment’s reliability. An unpaired *t*-test indicated (Table 3) that diabetes (*p* = 7.8 × 10^−8^), hypertension (1.0 × 10^−4^), and use of antithrombotic medications (0.4 × 10^−2^) were significantly correlated with eyelid swelling after blepharoptosis operation. According to regression analysis (Table 4), a large width of skin excision was most significantly associated with increased postoperative swelling (*p* = 1.35 × 10^−19^), followed by diabetes and aging (*p* = 1.52 × 10^−6^). Although the use of antithrombotic medication was identified as a risk factor, its influence was not particularly strong (*p* = 0.4 × 10^−2^). Factors such as hyperlipidemia (*p* = 0.30), width of aponeurosis advancement (*p* = 0.67), and MRD changes (*p* = 0.94) did not affect swelling.

**Table 3:** Swelling scores for *n* = 210 eyelids, 2 raters, total evaluations = 420.

Swelling score	0 No swelling	1 Trace swelling	2 Mild-moderate swelling	3 Severe swelling	Swelling score, Mean ± SD	*p*-value
Evaluated eyelids, number (%)	0	75 (17.9)	234 (55.7)	111 (26.4)	2.09 ± 0.66	0
Diabetes, number (%)	0	3 (3.9)	36 (47.4)	37 (48.7)	2.44 ± 0.57	7.8 × 10^−8^
Hypertension, number (%)	0	12 (7.7)	93 (59.6)	51 (32.7)	2.25 ± 0.59	1.0 × 10^−4^
Hyperlipidemia, number (%)	0	16 (23.5)	35 (51.5)	17 (25)	2.01 ± 0.70	0.30
Antithrombotic medications, number (%)	0	2 (5)	21 (52.5)	17 (42.5)	2.36 ± 0.59	0.4 × 10^−2^

**Table 4:** Regression analysis for *n* = 210 eyelids, 2 raters, total evaluations = 420.

Characteristics	Mean ± SD	*p*-value
Age	72.86 ± 10.0	1.52 × 10^−6^
Width of skin excision, mm	6.76 ± 3.36	1.35×10^−19^
Length of aponeurosis advancement, mm	9.29 ± 3.60	0.67
Surgical duration, min	45.63 ± 11.88	5.7 × 10^−8^
Intraoperative max SBP, mmHg	160.91 ± 21.73	9.49 × 10^−6^
Intraoperative max DBP, mmHg	78.06 ± 14.26	0.08
MRD change from pre- to 3 months post-operation, mm	2.01 ± 1.23	0.94

## Discussion

In this study, we identified patient- and surgery-related factors associated with early postoperative eyelid swelling in 105 patients. The goal is to identify such factors to better predict postoperative swelling in patients and implement preventive measures. To eliminate bias from the surgeon’s skill or preconceived notions, the surgeries were all performed by a single surgeon, and two independent plastic surgeons conducted the evaluations. The most significant factor contributing to early postoperative swelling was a larger width of skin excision; the presence of diabetes and older age were also found to have a significant impact. If both the surgeon and the patient can anticipate the potential for significant swelling, postoperative dissatisfaction or anxiety can be reduced while planning the postoperative schedule. Ideally, this data could also be used to develop strategies to prevent swelling, leading to improved outcomes for patients.

Among the most important principles when considering swelling is Starling’s law, which explains the factors contributing to edema including increased vascular permeability, elevated capillary pressure, and reduced lymphatic drainage. After surgery, edema increases primarily due to inflammation, elevated capillary pressure from increased local blood flow, and damage to the lymphatic system. Additionally, bleeding caused by microvascular damage contributes to further swelling. Notably, edema is defined as swelling caused by increased interstitial fluid within tissues or organs,^7^ whereas swelling can also arise due to bleeding or trauma.

By applying Starling’s law to the factors contributing to swelling in this study,^6^ it can be understood that removing a larger amount of skin increases tissue damage and inflammation, leading to increased vascular permeability and edema. Furthermore, diabetes, arteriosclerosis, and aging have been cited as causes of vascular endothelial damage that increase vascular permeability, which has been shown to increase the risk of edema. Incidentally, it is already well known that vascular permeability is elevated in diabetic patients with persistently high blood sugar levels.^8^^,^^9^ Thus, the eyelid swelling score is likely higher in diabetic patients for this reason. Additionally, it is believed that increased capillary pressure due to hypertension or intraoperative high blood pressure contributes to increased edema and bleeding.

Some studies have explored how to prevent swelling of the eyelid. Although cooling after surgery slightly reduces pain immediately after surgery, it does not reduce edema, erythema, or hematoma.^10^ A comparison of gauze pressure and transparent nonpressure eye shields after surgery showed no significant difference in edema or petechiae on the first and seventh days postsurgery.^11^ Most of the literature on measures to prevent eyelid swelling is related to rhinoplasty with open rhinoplasty, reporting that swelling can be reduced by intraoperative cooling,^12^ steroid administration,^13^ and tranexamic acid administration^14^ but it is unclear whether these measures can be applied after eyelid surgery.

Consumption of antithrombotic drugs can also play a role in swelling of the eyelid, but it has been found that their effect is not as strong as other factors. According to cardiovascular guidelines, surface surgery poses a low risk of bleeding, and surgery should be performed while the medication is administered.^15^ In dermatology, it has been reported that antithrombotic drugs do not increase the risk of bleeding during skin surgery^16^; a low risk of bleeding leads to less swelling. Therefore, we believe that it is not necessary to discontinue antithrombotic drugs during surgery, even if reducing swelling is a priority.

This study has several limitations. First, the relationship between the management of underlying conditions (such as blood sugar control, use of antihypertensive drugs, PT/APTT values, etc.) and swelling has not yet been fully clarified. Additionally, the influence of postoperative care variations on postoperative swelling has not been clearly established. It also remains unclear whether the timing and method used to assess eyelid swelling were appropriate. In particular, the clinical significance of swelling observed on postoperative day 7 is not understood. Further investigation is needed to identify additional factors that may help reduce postoperative swelling. Another limitation is the difficulty in identifying the exact cause of eyelid edema due to the contribution of multiple factors. In the future, increasing the number of cases and minimizing bias caused by confounding factors will be essential. Moreover, the method used to assess eyelid edema must be validated. Given challenges in quantitatively evaluating swelling, we relied on trained observers who visually assessed photographs as described in previous studies.^2^ However, this method may introduce a certain degree of bias, as visual assessments are subjective and rely on the observer’s experience and judgment, leading to some inevitable errors. In the future, we would like to consider using volumetric evaluation tools and 3D photographs. Single‐surgeon design: we recognize that conducting all procedures by a single surgeon enhances internal validity by minimizing technical variability, but may limit external generalizability. Furthermore, the mean age of the study population exceeded 70 years, and all included cases were limited to acquired ptosis. Given the age distribution of the cohort and the exclusion of patients with congenital ptosis or those requiring surgical approaches other than skin excision or aponeurosis advancement, the generalizability of our findings to younger populations or patients with congenital ptosis is limited.

## Conclusion

We retrospectively identified factors influencing early postoperative swelling in 105 patients with bilateral ptosis who underwent bilateral blepharoptosis surgery. Significant risk factors for early swelling included a larger width of skin excision, presence of diabetes, aging, hypertension, high intraoperative systolic blood pressure, and longer surgical duration. Identifying these risk factors before surgery can help reduce patient anxiety and dissatisfaction. Furthermore, developing preventive strategies that target these factors could help prevent postoperative swelling.

## Ethical approval

The study was approved by the National Hospital Organization Tokyo Medical Center Ethics Committee on March 27, 2024 (R23-106).

## Declaration of generative AI and AI-assisted technologies in the writing process

None.

## Data availability statement

The data that support the findings of this study are available from the corresponding author, Hiroko Ochiai, upon reasonable request.

## Funding

No author has a financial interest in any of the products, devices, or drugs described in this manuscript.

## Declaration of competing interest

None.
